# Supplementary material for: Dexmedetomidine Protects Cerebellar Neurons against Hyperoxia-Induced Oxidative Stress and Apoptosis in the Juvenile Rat
Source: Int J Mol Sci. 2023 Apr 25;24(9):7804. doi: 10.3390/ijms24097804 (PMC10178601; doi:10.3390/ijms24097804)
Supplement: Supplementary file 1 [file ijms-24-07804-s001.zip › Table S-1 Quantitation of CellDeath.pdf]

Table S-1 Quantitation of cell death

	NO	NOD	HY	HYD
**hyperoxia**	–	–	+	+
**dexmedetomidine**	–	+	–	+
		**P7**		
**Casp3+ ratio**	100±5.1	85±6.4	132±3.5	53±4.5
Casp3	100±7.9	105±5.2	142±9.6	148±8.3
AIF	100±7.7	101±6.7	146±8.1	99±5.8
		**P9**		
**Casp3+ ratio**	100±11.6	88±10.2	154±17.5	84±12.0
Casp3	100±6.2	86±5.6	144±4.8	88±7.6
AIF	100±9.9	110±5.7	122±9.0	101±8.8
		**P11**		
**Casp3+ ratio**	100±5.7	162±13.0	103±10.4	112±7.4
Casp3	100±7.2	147±5.7	164±8.0	109±3.0
AIF	100±6.8	110±5.0	119±6.0	90±6.5
		**P14**		
**Casp3+ ratio**	100±9.9	91±5.5	116±5.0	108±3.2
Casp3	100±7.4	99±7.2	102±5.4	72±1.1
AIF	100±8.1	105±6.4	92±3.2	74±6.8

Data are normalized to the level of rat pups exposed to normoxia at each time point (control 100 %). n = 6/group (ANOVA, Bonferroni's *post hoc* test).
