# Supplementary material for: Dexmedetomidine Protects Cerebellar Neurons against Hyperoxia-Induced Oxidative Stress and Apoptosis in the Juvenile Rat
Source: Int J Mol Sci. 2023 Apr 25;24(9):7804. doi: 10.3390/ijms24097804 (PMC10178601; doi:10.3390/ijms24097804)
Supplement: Supplementary file 1 [file ijms-24-07804-s001.zip › Table S-2 Quantification of oxidative stress.pdf]

Table S-2 Quantification of oxidative stress

	NO	NOD	HY	HYD
**hyperoxia**	–	–	+	+
**dexmedetomidine**	–	+	–	+
		**P7**		
**TBARS**	100±9.0	60±8.4	194±9.5	88±14.0
GCLC	100±8.4	100±4.3	147±13.8	117±4.6
Nrf2	100±7.9	106±6.9	152±10.8	83±11.0
SOD1	100±8.6	84±9.4	124±8.5	79±6.2
SOD2	100±6.3	83±6.6	132±9.9	98±2.4
SOD3	100±4.4	62±6.6	150±6.8	171±7.2
		**P9**		
**TBARS**	100±10.0	61±13.1	135±4.6	49±8.3
GCLC	100±8.6	106±6.3	106±4.6	95±9.0
Nrf2	100±3.9	96±19.2	137±9.8	75±10.2
SOD1	100±9.7	112±9.1	106±6.1	77±6.7
SOD2	100±7.7	136±7.7	146±9.4	126±15.5
SOD3	100±5.9	75±2.8	56±8.6	65±8.6
		**P11**		
**TBARS**	100±10.7	103±15.7	76±10.2	118±14.4
GCLC	100±11.8	209±26.1	656±90.8	385±20.6
Nrf2	100±8.6	114±5.9	115±1.8	80±5.6
SOD1	100±7.6	87±7.0	83±2.1	62±2.5
SOD2	100±4.2	106±4.9	132±5.6	105±2.7
SOD3	100±8.5	103±5.1	100±3.5	74±8.6
		**P14**		
**TBARS**	100±10.2	111±15.9	104±5.4	93±13.0
GCLC	100±6.0	108±9.4	82±5.1	72±6.2
Nrf2	100±8.2	81±7.0	73±3.2	63±7.8
SOD1	100±5.5	87±3.3	78±5.2	71±4.1
SOD2	100±5.3	116±5.2	97±4.7	93±5.5
SOD3	100±3.8	118±4.7	116±7.3	92±9.1

Data are normalized to the level of rat pups exposed to normoxia at each time point (control 100 %). n = 6/group (ANOVA, Bonferroni's *post hoc* test).
