# Supplementary material for: Dexmedetomidine Protects Cerebellar Neurons against Hyperoxia-Induced Oxidative Stress and Apoptosis in the Juvenile Rat
Source: Int J Mol Sci. 2023 Apr 25;24(9):7804. doi: 10.3390/ijms24097804 (PMC10178601; doi:10.3390/ijms24097804)
Supplement: Supplementary file 1 [file ijms-24-07804-s001.zip › Table S-3 Quantification of inflammation.pdf]

Table S-3 Quantification of inflammation

	NO	NOD	HY	HYD
**hyperoxia**	–	–	+	+
**dexmedetomidine**	–	+	–	+
		**P7**		
**TNFα**	100±9.2	107±6.4	137±7.4	90±10.6
TNFα	100±11.9	69±2.7	192±23.6	83±7.5
IL1β	100±6.0	51±9.3	62±6.4	72±5.0
		**P9**		
**TNFα**	100±9.4	91±11.7	99±8.0	90±8.0
TNFα	100±6.3	107±9.4	130±6.6	82±6.5
IL1β	100±6.9	106±7.8	79±11.5	55±6.8
		**P11**		
**TNFα**	100±12.8	110±9.6	107±10.3	190±15.5
TNFα	100±7.5	88±7.6	55±4.3	158±5.8
IL1β	100±7.9	95±8.2	83±9.8	74±8.3
		**P14**		
**TNFα**	100±9.5	114±6.9	114±9.7	111±10.2
TNFα	100±6.1	91±5.5	86±0.7	90±7.9
IL1β	100±4.9	73±4.5	60±9.1	83±8.8

Data are normalized to the level of rat pups exposed to normoxia at each time point (control 100 %). n = 6/group (ANOVA, Bonferroni's *post hoc* test).
